# Seeking digital maternity healthcare during the pandemic health system shock: a systematic review of women's experiences in low- and middle-income countries

**DOI:** 10.3389/frph.2025.1734456

**Published:** 2026-01-12

**Authors:** Tisha Dasgupta, Emily Russell, Camila Carbajal, Gillian Horgan, Lili Peterson, Hiten D. Mistry, Rachael Buabeng, Milly Wilson, Valerie Smith, Harriet Boulding, Kayleigh S. Sheen, Aricca D. Van Citters, Eugene C. Nelson, Emma L. Duncan, Peter von Dadelszen, The RESILIENT Study Group, Laura A Magee, Debra E Bick, Harriet Boulding, Kathryn Dalrymple, Tisha Dasgupta, Emma L Duncan, Abigail Easter, Julia Fox-Rushby, Gillian Horgan, Asma Khalil, Alice McGreevy, Hiten D Mistry, Eugene C Nelson, Lucilla Poston, Paul Seed, Sergio A Silverio, Marina Soley-Bori, Florence Tydeman, Aricca D Van Citters, Sara L White, Ingrid Wolfe, Yanzhong Wang, Peter von Dadelszen, Sergio A. Silverio, Laura A. Magee

**Affiliations:** 1 Department of Women & Children’s Health, School of Life Course & Population Sciences, King’s College London, London, United Kingdom; 2 Department of Population Health Sciences, School of Life Course & Population Sciences, King’s College London, London, United Kingdom; 3 The RESILIENT Study Patient & Public Involvement & Engagement Advisory Group, United Kingdom; 4 Mummy’s Day out, London, United Kingdom; 5 School of Nursing, Midwifery & Health Systems, College of Health and Agricultural Sciences, University College Dublin, Dublin, Republic of Ireland; 6 The Policy Institute, Faculty of Social Science & Public Policy, King’s College London, London, United Kingdom; 7 Department of Social Sciences, College of Health, Science and Society, University of the West of United Kingdom Bristol, Bristol, United Kingdom; 8 The RESILIENT Study Technical Advisory Group, United Kingdom; 9 The Dartmouth Institute for Health Policy & Clinical Practice, Geisel School of Medicine, Dartmouth College, Lebanon, NH, United States; 10 Department of Twin Research & Genetic Epidemiology, School of Life Course & Population Sciences, King’s College London, London, United Kingdom; 11 The RESILIENT Study Group, United Kingdom; 12 Department of Psychology, Institute of Population Health, University of Liverpool, Liverpool, United Kingdom

**Keywords:** COVID-19, digital health, LMIC, maternity care, pregnancy, qualitative research, systematic review, women

## Abstract

**Background:**

The pandemic created global disruption acting as a health system shock not seen before in living memory. As a consequence, there were significant implications for healthcare delivery in low- and middle-income countries. Challenges such as lockdown restrictions created substantial modifications to the delivery of maternity care. This review aims to explore the experiences of maternity care by women, specifically in low- and middle-income countries, during the pandemic global health system shock.

**Methods:**

A systematic search was conducted for qualitative literature published about maternity healthcare experiences during the pandemic. Studies which provided qualitative data on women's experiences of digital healthcare, and other maternity care reconfigurations in low- and middle-income countries were included. The studies underwent quality assessment using twelve criteria adapted from the quality appraisal tool developed by the Evidence for Policy & Practice Information (EPPI) Centre. Thematic synthesis was employed.

**Results:**

Of the 21,860 records identified, 30 met the inclusion criteria for this review. Across the 4 key predetermined areas of study: (1) Care seeking and experience; (2) Digital health; (3) Vaccination; and (4) Ethical future of maternity services; 10 concepts were reported upon, namely: (1.1) Emotional challenges and uncertainty, (1.2) Disruption of services, (1.3) Stigma and discrimination, and (1.4) Changing support systems; (2.1) Safety and reassurance, (2.2) Locus of responsibility; (3.1) Vaccine understanding and acceptance; and (4.1) Improvements for maternity care delivery, (4.2) Implementation of virtual care, (4.3) Education and empowerment.

**Conclusion:**

Our findings suggest emotional challenges, isolation, and limited access to maternity services were prominent among pregnant individuals in low- and middle-income countries. This synthesis provides insights into how pandemic associated adaptations, which have been retained beyond, such as digital health solutions were experienced by women within constrained health systems, revealing both opportunities and persistent gaps in digital health access and equity. Although a review of low- and middle-income countries—there is learning to be taken from these settings which could easily be applied not only across low- and middle-income countries, but also in high-income settings, in the form of reverse (or “trickle-up”) innovation to improve maternity care as we recover and re-build from the pandemic and offer more resilient ways of providing maternity care through future health system shocks.

**Systematic Review Registration**: https://www.crd.york.ac.uk/PROSPERO/view/CRD42022355948, identifier CRD42022355948.

## 1 Introduction

The COVID-19 pandemic created widespread shock across health systems globally. The burden of disease was borne by those in low- and middle-income countries (LMICs), disproportionally, where ∼84 million cases and >1 million deaths were reported by May 2024 (1). As an essential service, maternal healthcare remained available to women throughout the pandemic, although the care offered was reconfigured and resources were re-allocated to support infection control strategies (2).

However, the service delivery changes implemented during this period have continued post-pandemic and remain critical to evaluate.

Across the world, maternity care was transitioned to remote provision (3), but implementation was hindered by a shortage of doctors, poor infrastructure, low technological literacy, and digital poverty (4). Fear of contracting COVID-19 and government lockdowns meant women were unlikely to attend for the eight recommended antenatal care (ANC) visits (5). Appointments were often postponed, or the availability of antenatal testing was reduced, causing distress (6). Some women attended clinics only in their third trimester, when care was deemed absolutely necessary, thereby missing early opportunities for essential health promotion such as vaccination (5). Most hospitals prohibited or severely restricted visitors, and a lack of labour support caused anxiety and an isolating and negative birth experience (6, 7). Additionally, there were reports of pregnant women not attending ANC at all and choosing to deliver at home (5). Although by December 2020, multiple variations of the COVID-19 vaccine were approved for global use, access and uptake remained low in many LMICs (8), related to inequitable distribution, norms around vaccine acceptance, geographical barriers and poor access to immunisation facilities, and logistical issues of maintaining a cold chain for vaccine storage (9).

A global systematic review synthesised data from 48 studies on women's and healthcare professionals' (HCPs') experiences of maternity care receipt or delivery during the COVID-19 pandemic (10). Women experienced heightened levels of anxiety, uncertainty, and isolation during pregnancy. The prominent adoption of telemedicine offered both benefits and challenges, but was deemed ineffective as a substitute for in-person care and particularly in LMICs, language barriers and digital illiteracy were notable challenges (10). However, only six of these studies included were undertaken in LMICs, and there was little discussion of any differences in findings between high-income countries (HICs) and LMICs (10).

The aim of this review is to update the above by Flaherty et al., to explore experiences of maternity care, in LMICs, and how they may have changed since June 2021, to inform future planning of maternity services. The review forms part of the work of The RESILIENT Study (11) of post-pandemic planning for maternity care, with key concepts of service delivery: care seeking and experience, digital health including virtual care and self-monitoring, COVID-19 vaccination, and an ethical future of maternity services.

## 2 Methods

This Systematic review is part of a wider RESILIENT: Post-pandemic planning of maternity services programme of work (11) which is registered with PROSPERO [CRD42022355948] (12) and complies with PRISMA guidelines (see Supplementary Table S1) (13).

### 2.1 Inclusion criteria

Our inclusion criteria followed the SPIDER (Sample, Phenomenon of Interest, Design, Evaluation, Research type) framework, as in the original systematic review (10). The **S**ample comprised women and birthing people (hereby, simply women) of all parity who were either planning pregnancy, pregnant, or up to six months postpartum at the time of the study. The **P**henomenon of **I**nterest focused on experiences of maternity care during the COVID-19 pandemic; maternity care encompassed antenatal, intrapartum, and postnatal care, delivered in all settings, including hospitals, at home, or in the community. Studies related to monitoring and assessment of health and wellbeing, and vaccinations were also included. Study **D**esigns included were qualitative or mixed-methods studies, wherein qualitative data had been formally analysed. **E**valuation centred on capturing the perspectives and experiences of maternity care during the pandemic. The **R**esearch included literature published in LMICs and full-texts available in English.

### 2.2 Search strategy and selection

Electronic databases of Scopus, MEDLINE, EMBASE, CINAHL, PsycINFO, and the Cochrane COVID study register were systematically searched. Search terms used were replicated from the original global review (10) (see Supplementary Table S2). We extended the search dates to include studies published between June 2021 and October 2022 and further updated to June 2024. Duplicates were identified and removed using EndNote referencing software. Records were uploaded to Rayyan, an internet-based systematic screening tool. All titles/abstracts and full-text articles were screened in duplicate by at least two independent members of the RESILIENT review team. Reviewers worked independently within Rayyan at both stages. Any disagreements at the title/abstract and full-text stage were discussed during regular team meetings and resolved by consensus within the screening team. Disagreements were resolved in regular team meeting discussions, by consensus of the wider team members. A decision was made *a priori* to divide the large volume of identified records by geographic location (UK, other HICs, LMICs) and participant type (women, HCPs) in alignment with the aims of the RESILIENT study.

### 2.3 Quality assessment

As used by Flaherty et al. (10), a quality assessment tool developed by the Evidence for Policy and Practice Information Centre was used to assess the methodological rigor of included studies (14). Each study underwent evaluation by a reviewer (ER) against the 12 assessment criteria within the appraisal tool. Independent second evaluation was conducted for 20% of the studies (by TD). These criteria facilitated an appraisal (as yes, no, or partial) of the adequacy of study reporting, the quality of study methods, and both the reliability and validity of data collection and analysis.

### 2.4 Data extraction and synthesis

Data extraction was conducted using a pre-designed form in Microsoft Excel. In addition to documenting whether each study addressed any of the three main maternity service reconfigurations of particular interest to RESILIENT (i.e., virtual care, self-monitoring, and vaccination), the form included: study objectives, participant characteristics, study setting, data collection period, methods, data collection and analysis methodology, and themes reported by the authors.

Synthesis of qualitative data was conducted using Thematic Synthesis (15) adapted by coding into the RESILIENT concepts: (1) Care-seeking and experience, (2) Digital Health: Virtual care and Self-monitoring, (3) Vaccination, and (4) Ethical future of maternity care. Abstracted data from Discussion sections were broadly coded into these four categories, and then line-by-line coded to inductively generate descriptive themes. Codes were compared for conceptual similarity, clustered into preliminary groups, and then iteratively refined into descriptive themes, the latter in discussion with the wider study team. Data were extracted and synthesised independently by ER, and then by a second, independent investigator (TD) for 20% of included studies. Disagreement was resolved by consensus with the wider team, at regular meetings, which included discussion with the RESILIENT study PPIE group. Results sections were not synthesised to prevent rendering circular logic, which could occur if we synthesized the same primary data that authors had already interpreted and presented in their results sections, essentially double-counting or reinforcing the same evidence through multiple lenses. By focusing on Discussion sections, we aimed to extract higher-level interpretations and conceptual insights rather than duplicating raw data.

## 3 Results

### 3.1 Search and selection

**Figure 1:**
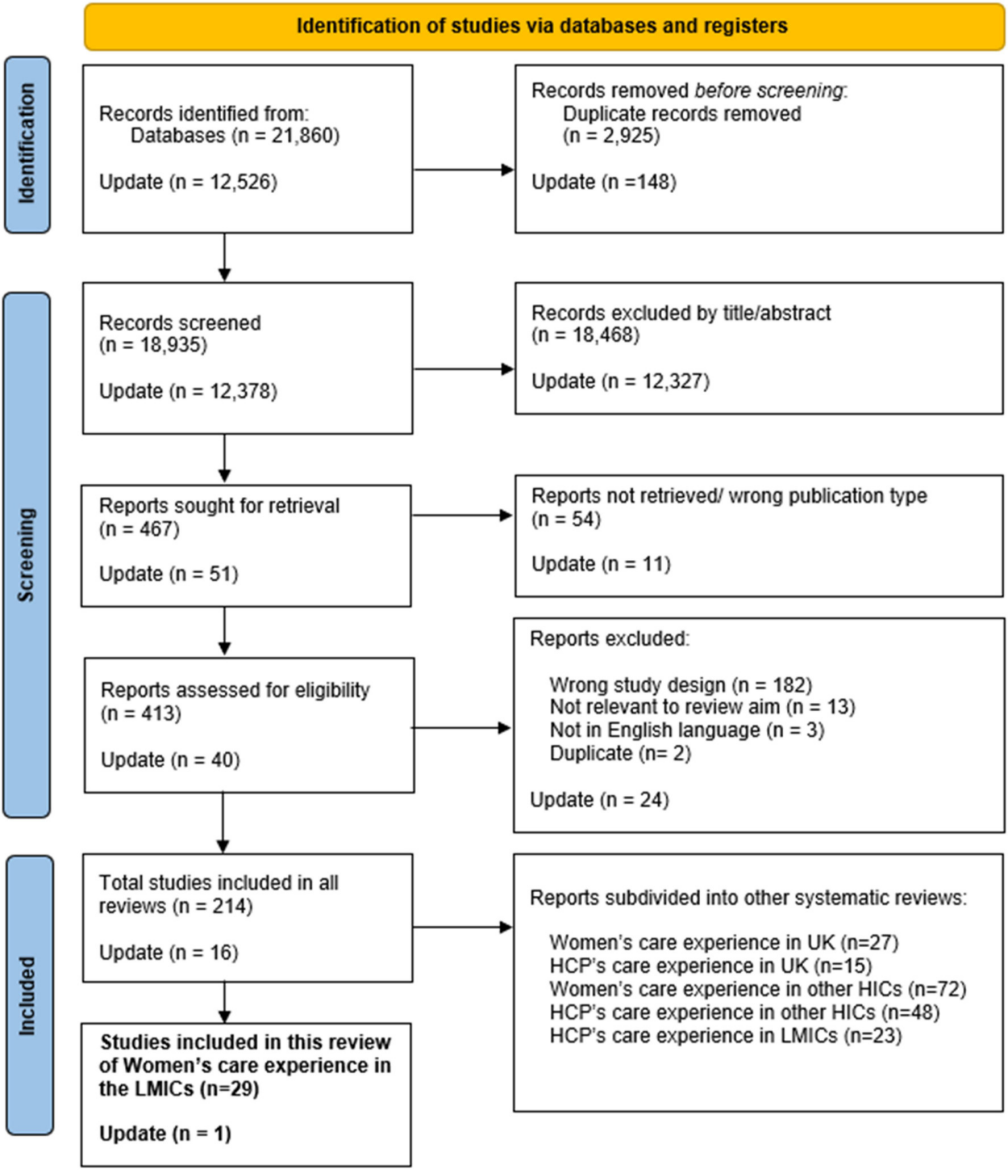
PRISMA flow diagram: study selection process.

Of 21,860 records identified, 213 met our inclusion criteria, of which 29 studies evaluated women's experiences of maternity care during the pandemic in LMICs and were included in this review. An updated search found an additional 16 studies for inclusion in total, and one which met the focus of this specific review; in total 30. Figure 1 presents details of the search and selection process, includingdivision of the other studies by geographic location (UK, other HICs, and LMICs) and population group (service users, HCPs), addressed in reviews that have been published (16, 17) or are in progress.

### 3.2 Description of included studies

The 30 studies (6, 18–45) included data from over 600 women from 17 LMICs. Supplementary Table S3 presents key details of each study. In brief, studies were conducted between March 2020 and October 2021. Most (*n* = 26) studies utilised semi-structured, in-depth interviews to gather data (5, 6, 18–19, 21–23, 25–27, 29, 30, 32–45), with two studies collecting additional data using field diaries or demographic surveys (21, 41). One study undertook a survey by telephone (20), one conducted focus group discussions (24), another facilitated virtual culture circles (31), and one study used narrative interviews (28). Analyses were conducted most frequently using thematic analysis (*n* = 12) (5, 18, 21, 22, 27, 32, 35, 36, 39, 42, 44, 45). Most (*n* = 28) (6, 18–42, 44, 45) reported on care-seeking and care-experience which included mental health; six (24, 29, 30, 38, 39, 41) on digital health including virtual care delivery and self-monitoring; two (28, 43) on vaccination; and 22 (6, 18–20, 22, 25–35, 37–41) on ethical future of maternity care.

### 3.3 Quality assessment

All except one study were moderate-to-high quality, meeting all (25, 27, 33–35, 44) or 11/12 criteria (18, 19–22, 29, 31, 32, 36–38, 41, 45). Failure to meet the twelfth criterion was usually due to the lack of active involvement of participants in the study design (see Supplementary Table S4). Although no studies were excluded on the basis of quality, the appraisal process informed our interpretation by allowing us to consider the strength and consistency of evidence contributing to each theme, and to interpret findings from the single lower-quality study with appropriate caution.

### 3.4 Synthesis and findings

Data synthesis yielded 10 themes under the four RESILIENT concepts as presented in Table 1. Key excerpts from original texts are presented in Tables 2–5 in support of synthesised findings.

**Table 1:** Process of theme development.

Concept	Theme	Codes
1. Care seeking and experience	1.1 Emotional challenges and uncertainty	Guilt or believing did something wrong
		Uncertainty and fear of getting infected/ health facilities
		Lack of knowledge and concern for fetus
	1.2 Disruptions of services	Difficulty accessing healthcare
		Change of care
		Compromised care
	1.3 Stigma and discrimination	Disease associated stigmatisation
		Discrimination
		Harassment
	1.4 Changing support systems	Reduced shared experience
		Lack of support
		Positive Support
2. Digital Health	2.1 Safety and reassurance	Virtual options improving maternity care
		Increase in virtual care
	2.2 Locus of responsibility for monitoring health	Self-monitoring
3. Vaccination	3.1 Vaccine understanding and acceptance	Lack of availability
		High vaccine hesitancy
4. Ethical future of maternity care	5.1 Improvements for maternity care delivery	Change in management of care
		Future regarding pandemics
		Protection of vulnerable women
	5.2 Implementation of digital health virtual care	Social networking groups
		Improving challenges of delivering maternity care during crisis
	5.3 Education and empowerment	Coping with adversity
		Education on disease/COVID-19
		Health Education on pregnancy
		Empowerment during peripartum period

**Table 2:** Concept 1: care-seeking and care experience.

Themes	Quotations
1.1 Emotional challenges and uncertainty	“Guilt was a recurring emotion for them because they held themselves responsible for their own and their fetus's health during COVID-19” (38).
	“Some experienced “anxiety and fear” because of the risk of virus transmission to their fetus, “great sadness” because of their relatives being COVID- 19 positive or passing away, and “depression” because of lack of social support and loneliness” (44).
	“expectant mothers sought information from all available sources to reassure themselves about their pregnancy progress and foetal wellbeing. They sought information from non-official electronic sources, used their previous experience of pregnancy, and their knowledge if they were nurses themselves” (21).
1.2 Disruptions of services	“Barriers to accessing MNCH services by patients in this study included fear of contracting COVID-19 at health facilities, lack of funds to pay for services at the health facilities, transportation difficulties, shortage of manpower, long waiting times and a daily capped number of patients to be attended to at the hospitals, negative attitude of healthcare workers, harassment by security agents, and stigmatization of service users by health workers” (22).
1.3 Stigma and discrimination	“Respondents felt as though the pandemic, and the myriad of protective measures it brought, created conditions for authority figures to actively act on their prejudices based on caste and class, in the guise of enforcing social distancing guidelines” (42).
1.4 Changing support systems	“Our findings showed that unconditional support from husbands, families and communities immensely helped these women maintain their commitment to breastfeeding during this pandemic” (36).
	“central aspects of the transition to motherhood, such as the shared experience of pregnancy, childbirth, and puerperium—so important for Brazilian mothers—were blocked, leading to a deep feeling of helplessness and of disintegration of transgenerational bonds” (28).

MNCH, Maternal and newborn child health.

**Table 3:** Concept 2: digital health.

Sub-theme	Quotation
2.1 Safety and reassurance	“Telephone, e-mail, and video consultations increased significantly in primary care during the coronavirus pandemic… They felt secure receiving phone calls from medical doctors” (41).
	“Outpatient teleconsultation services, availability of tertiary and maternity institutes, and well-functioning primary care centres in the study setting could be the reasons for a relatively better ANC provision during the pandemic” (29).
2.2 Locus of Responsibility for monitoring health	“The participants' expectations regarding the methods for monitoring the fetus' condition focused on the use of the latest technology that was easy to apply, simple, and familiar, like cell phones. The results indicated that the participants need a fetal monitoring tool that can be used independently” (24).

**Table 4:** Concept 3: vaccination.

Theme	Quotation
3.1 Vaccine understanding and acceptance	“The main reason for this low COVID-19 vaccine acceptance of the mothers might be rumours on COVID-19 vaccine; they fear the vaccine adverse effect and mothers have no clear information about the side effects and the complication of the vaccine” (43).
	“assisted by a precarious public health system that neglected women's health and that, despite the high number of maternal deaths—the highest in the world—for a long time did not make vaccination available to pregnant and postpartum women” (28).

**Table 5:** Concept 4: ethical future of maternity care.

Themes	Quotation
4.1 Improvements for maternity care delivery	“The results of the present study reported that all the pre-birth as well as post-birth respondents suggested to improve the maternal care services by establishing a database of gynaecologists, providing birth plan and appropriate guidance/counselling to the new mothers regarding danger signs, miscarriage, lifestyle modification, lactation, post-partum depression, birth signs and complications associated with pregnancy as well as delivery” (39).
	“MCH-related emergency care should prioritize improving access to health services by strengthening community-based antenatal care” (30).
4.2 Implementation of digital health and virtual care	“It is recommended that mechanisms such as digital platforms, mom-connect, telehealth can be used to assist pregnant women during a crisis such as pandemics or unrest in the country that may prohibit them to attend antenatal care” (26).
	“With some pre-planning, greater investment in enabling technologies [telemedicine, online counselling, and virtual reality (VR) technology] to help connect and advise women experiencing pregnancy, and some targeted financial support, the challenges could be alleviated” (27).
4.3 Education and empowerment	“Continuous efforts should be made to empower women and to generally improve their psychological well-being, so they will become more successful in coping with adversities” (6).
	“Thus, the importance of a consolidated maternal knowledge about the disease is revealed, in order to favour the development, the reduction of vulnerability and the protection of the child in its microsystem” (32).

#### 3.4.1 Concept 1: care seeking and experience

The first concept comprised five themes: emotional challenges, disruption of services, stigma and discrimination, feelings of anxiety and uncertainty, and changing support systems (Table 2). All but one study contributed data for this theme (6, 18–42, 44).

##### 3.4.1.1 Emotional challenges and uncertainty

The emergence of the pandemic, accompanied by lockdowns, limited access to care, and additional restrictions further heightened emotions. As global cases of COVID-19 continued to rise, mothers feared for both themselves and their babies (21), often afraid to attend hospital appointments (22, 26, 42, 45). Despair and depression were felt in response to losing loved ones and feeling isolated (18, 23, 44). Women grappled with feelings of guilt or uncertainty, harbouring concerns that they were jeopardizing their babies' health, by giving birth during the pandemic (36, 38, 41, 45).

Women experienced anxiety and uncertainty when they encountered misinformation and myths circulated online and within their communities (19, 33, 34, 41, 42). Some women sought information from all possible sources to reassure themselves (20, 21). Many individuals took proactive measures to mitigate their risk of transmission by adhering to health protocols, such as hand-washing and mask-wearing (21, 25, 34), although some found this difficult, such as during childbirth (26).

##### 3.4.1.2 Disruption of services

Antenatal classes were cancelled, and many women faced challenges in accessing regular antenatal check-ups, in private and public clinics (6, 26). Fear of contracting the virus led to reduced attendance at appointments or no attendance at all, exacerbating poor health outcomes (23, 29, 38, 44). Public healthcare systems redirected personnel and resources to urgent treatment of COVID-19 (28, 33, 35). Some women sought recourse at private healthcare facilities, although job losses and the economic downturn meant that some women were unable to afford this, or sought financial assistance to do so, including borrowing money (22, 28, 33, 35, 45).

Nevertheless, there were some positive findings. Continued provision of community-based care made routine immunisation services and antenatal check-ups relatively easy for certain communities in India (30). Some women reported receiving supportive and empathetic care from HCPs, which helped improve their overall experience of maternity care during the pandemic (38).

##### 3.4.1.3 Stigma and discrimination

This sub-theme covered studies which reported on stigmatisation and discrimination due to structural factors such as religion or immigration status, as well as positive COVID-19 status. In six studies (5, 18, 22, 34, 41, 42) stigmatisation was described for those who were COVID-19 positive; this was by HCPs and community members, and there were reports of physical, verbal, and even sexual abuse. Strict lockdown restrictions in some countries (e.g., Nigeria), led to harassment by law enforcement when women attended hospitals (22). Together, these practices resulted in some women avoiding necessary treatment (34).

Additionally, discriminatory policies against pregnant Muslim and migrant women exacerbated existing intersectional inequalities (5).

##### 3.4.1.4 Changing support systems

Most women faced altered support systems, which had negative and positive impacts on maternal well-being. In some cases, being forced to stay home during lockdown strengthened and improved family relationships (6). Family and community support emerged as crucial sources of resilience, to cope with challenges (41) and to maintain breastfeeding, specifically (36). However, other studies reported reduced support during lockdown, including informal and formal social support, and increased social isolation worsened family stress (18, 32, 45). Restrictions on partners and support members during ANC, and labour and delivery in hospitals, hindered shared experiences, and deprived mothers of the traditional support integral to maternal well-being and recovery in many communities (6, 28).

#### 3.4.2 Concept 2: digital health

Only six (24, 29, 30, 38, 39, 41) of the 30 studies contributed to the concept of digital health utilisation; of which five (29, 30, 38, 39, 41) were centred around increased virtual care delivery which was mostly viewed positively. Notably, none of these five studies specifically investigated virtual delivery of care as a research aim. Only one study addressed self-monitoring including using home-based digital technology, primarily for fetal monitoring of heart rate and movement (24). The key point of this study was shifting locus of responsibility for monitoring health. Details in Table 3 below.

##### 3.4.2.1 Safety and reassurance

Virtual care rose substantially in use (41), and online consultations were promoted (30). Virtual care delivery was felt to be effective in providing support and reassurance to pregnant women, including those infected with COVID-19 (29, 30, 41). Both in-person and online care modalities were reported to create a sense of positive well-being among some individuals with high-risk pregnancies (38).

On the other hand, some studies recognised the lack of access to these technologies and how their implementation could provide benefits for pregnant women (31, 33).

##### 3.4.2.2 Locus of responsibility for monitoring health

Within the context of growing self-monitoring, digital tools and virtual care can inadvertently shift expectations toward individuals by requiring them to track symptoms, navigate digital platforms, and make decisions that previously involved direct support from HCPs. While virtual care provided remote access to professional support, some women also began taking on a more active role in monitoring their own health during the pandemic, supported by technology. Participants reported employing both manual monitoring methods and using various tools, such as measurement bands (24). They expressed a desire for self-fetal monitoring that was accessible, with user-friendly technology which could be used independently (24).

#### 3.4.3 Concept 3: vaccination

Only two studies addressed this concept (28, 43), with data centred around understanding and acceptance of the vaccine (Table 4).

##### 3.4.3.1 Vaccine understanding and acceptance

A study conducted in Ethiopia centred around vaccine acceptance, finding only a small number of individuals who accepted the vaccine; most expressed fears of potential side effects, but those who were more likely to accept the vaccine felt properly-informed, were older, or had medical or other chronic conditions (43). In another study, the Brazilian health system was criticised for the delay in making the COVID-19 vaccine available to those who were pregnant or postpartum, despite the high maternal mortality rate (28).

#### 3.4.4 Concept 4: ethical future of maternity care

The concept of an ethical future of maternity care encompasses the collective vision for advancing maternal healthcare and building back services in a post-pandemic world, with a focus on alleviating inequalities. Twenty-two studies contributed data to this concept (6, 18–20, 22, 25–35, 37–41), with three themes: improvements for maternity care delivery, implementation of digital health, and education and empowerment (Table 5).

##### 3.4.4.1 Improvements for maternity care delivery

Suggested improvements to maternity care included implementation and strengthening of community-based programs, such as home visits and ANC within local communities (5, 26, 30, 35). HCPs were regarded as needing to both provide evidence-based information, and take an active role in promoting awareness about misinformation, on television or online (18). At the health system level, advocacy was felt to be crucial for pregnant women who are refugees, particularly for their inclusion in health policies and to combat discriminatory practices (5). Striking a balance between the two was considered important to minimise the spread of infectious disease (COVID-19 or future) preservation of cultural norms and practices (20).

##### 3.4.4.2 Implementation of digital health and virtual care

Maximising the use of digital applications and web-based platforms was viewed as a key strategy for the future (26, 39). Implementing virtual booking appointments, including screening for poor mental well-being, and providing healthcare workers with infection control training was emphasised (19, 23). Internet-based groups linking service users and HCPs offers an opportunity for education and distribution of advice, particularly in times of crisis (19). Additionally, virtual care has the potential to provide care to isolated patients in rural areas, improving accessibility and flexibility of routine services (27, 28, 38). Televising ANC classes is an option for providing information in low-resource settings, if other forms of online learning are not possible (20).

##### 3.4.4.3 Education and empowerment

Sustained health education efforts could help in dispelling myths and misconceptions surrounding the pandemic or any future novel disease (22, 26). Social media can be harnessed to provide accessible and interactive information (25). Furthermore, training pregnant women in relaxation techniques can aid in reducing anxiety, enabling them to better cope with adversity, and foster resilience (40). When women are empowered to actively monitor their health and well-being, it can lead to a better pregnancy experience and better communication with HCPs about their needs and concerns (40).

## 4 Discussion

### 4.1 Main findings

To the best of our knowledge, this is the first systematic review synthesising women's qualitative experiences of maternity care during the COVID-19 pandemic, focused solely on LMICs. It collates information from 30 studies spanning 17 countries, building on Flaherty et al.'s review (10) which included data from only six studies in LMICs, by adding research published in the latter half of the pandemic, and of digital health interventions which were widely implemented at the time, albeit limited. Ten themes were identified across four main concepts, namely: (1) Care seeking and care experience, with subthemes of: (1.1) Emotional challenges and uncertainty, (1.2) Disruption of services, (1.3) Stigma and discrimination, and (1.4) Changing support systems; (2) Digital health: (2.1) Safety and reassurance, (2.1) Locus of responsibility; (3) Vaccination: (3.1) Vaccine understanding and acceptance; and (4) Ethical future of maternity care: (4.1) Improvements for maternity care delivery, (4.2) Implementation of virtual care, (4.3) Education and empowerment. This synthesis provides insights into how pandemic-associated adaptations such as digital health solutions were experienced by women within constrained health systems, revealing both opportunities and persistent gaps in access and equity.

### 4.2 Comparison with the literature

Although the pandemic accelerated implementation and usage of digital health modalities globally, only six studies reported on this experience. This contrasts with our congruent reviews of experiences in the UK (16, 17), where over 80% of studies contributed data to this theme. This under-representation demonstrates the persisting digital divide between HICs and LMICs (46). Nonetheless, the discussions were generally positive, finding telemedicine an effective tool in LMICs for providing maternity services during the pandemic, offering increased convenience, reassurance and safety. We also report that women expressed a desire to perform more self-monitoring in the future, suggesting the use of user-friendly digital applications (25). Nevertheless, there are many barriers to implementation of self-monitoring. A study with obstetricians in Ghana strongly endorsed use of home blood pressure monitoring in pregnancy (47), but identified that the cost of the monitors, absence of a communication system for conveying values, and the necessity for patient education were challenges encountered (47). Others have express concerns for equitable participation in digital health, citing structural barriers such as limited internet access, economic impacts, and privacy concerns (48, 49). Whilst digital interventions have been found to be useful particularly for remote communities, there are limitations imposed by connectivity. A United Nations report from 2021 found that while 75% of people in the least-developed countries were covered by a broadband network, only a quarter of them were actually connected (50); this divide was attributed to cost, a lack of awareness of what services were available, and a lack of digital skills (50). Any implementation of virtual care in LMICs must be cautious not to exacerbate health inequities. Furthermore, while digital health interventions may improve care attendance, the impact on pregnancy outcomes has been variable (51) and costs to the health system in LMICs during the pandemic remain unexplored and needs further research.

We show that woman continued to face significant emotional challenges during the pandemic, particularly characterized by fear and uncertainty. Disruption of maternity services has been found to be associated with decrease in utilisation of critical services during this period (52, 53). Both pregnant women and their partners were left feeling lonely, overwhelmed, stressed and vulnerable (16, 53–57). Some women found that social support from family and friends, albeit limited, provided an invaluable source of resilience, as coping mechanisms which included personal (for e.g., meaning-making), relational (support from partner), and contextual (social connectedness) themes (58). Wider literature recommends strategies to build back robust services by incorporating mental health into plans for universal coverage, integrating assessments into routine care pathways such as during ANC, and leveraging digital technologies (59). However, there remains a risk for this to be underutilised due to infrastructural and literacy constraints (60), reinforcing the need for resource-mindful and context-appropriate mental health solutions (61) that do not overly burden women with the onus of responsibility over their health.

This review highlights a finding not evident in our other work (16) of stigma and discrimination faced by many mothers during the pandemic, particularly if they were COVID-19 positive or from marginalised backgrounds. Other research cites lack of information, misconceptions, and fear of the virus as the root cause for stigma. Experiential knowledge from previous infectious diseases (e.g., HIV/AIDS and Ebola), shows that disease-related stigma can often act as a barrier in controlling disease spread and equitable development in LMICs (62). Digital exclusion and illiteracy could have compounded these experiences as lack of knowledge about viral transmission of coronavirus and potential harm to the baby, often fuelled by misinformation and exacerbated by social media, has also been reported in other studies globally (53, 63, 64).

We found only two studies reporting on COVID-19 vaccination for pregnant women in LMICs (26, 43). This raises pertinent questions about this research gap. Global COVID-19 vaccine inequity has been attributed to globally inequitable distribution of vaccines (65, 66); constraints in manufacturing capacity along with procurement and storage mechanisms (67, 68), vaccine hesitancy; and LMICs being less likely to have in place guidelines for vaccination in pregnancy (66); mixed evidence exists in regards to COVID-19 vaccine hesitancy in LMICs, with evidence showing both higher (69) and lower (68) levels of acceptance when compared to HICs. While digital platforms have the potential to support far-reaching and equitable communication about vaccination and tracking utilisation, inconsistent infrastructure and low digital literacy as would limit their effectiveness.

Finally, our review shows that digital transformation of maternity care in LMICs must not replicate systems from HICs, but rather prioritise the importance of cultural context, inclusivity, ethical design, and sustainability. Community-based services have been persistently lauded as a strategy to improve maternal and child health outcomes in LMICs (30, 70) which can be substantiated with digital health modalities integrated into existing care pathways in an innovative manner. Alongside national-level improvement and investment into digital infrastructure, our review suggests localised scaling-up of virtual care to improve accessibility of care through remote consultations, online mental health assessments, televised ANC classes, improved communication of up-to-date safety evidence and guidance, and networking platforms bringing together HCPs and service users to offer advice or answer simple questions (19, 20, 37, 39). These are coherent with the WHO guidelines for global strategy on digital health which calls for national digital health strategies aligned with health goals, strengthening data governance structures, and prioritising people-centred and inclusive interventions enabled by digital health (46).

These findings have several policy implications. The emotional challenges and heightened anxiety reported across multiple studies (Concept 1, themes 1.1 and 1.4) underscore the need for routine integration of mental health support within maternity services. Likewise, widespread disruptions to antenatal and postnatal care (Concept 1, theme 1.2) highlight the importance of strengthening community-based and flexible care delivery models that can withstand future system shocks. The predominantly positive reception of virtual care alongside women's emerging interest in self-monitoring (Concept 2), points to the potential of hybrid care models, provided that issues of access and digital literacy are simultaneously addressed. The limited but concerning evidence surrounding vaccination understanding and uptake (Concept 3) emphasizes the need for clear and consistent public health communication and education campaigns. The findings of Concept 4: ethical future of maternity care reflect these recommendations.

### 4.3 Strengths, limitations, and future directions

This review benefits from robust systematic review methodology, including data extraction and synthesis. Our focus on LMICs is a unique element of this work. The decision to only include studies published in English may have unknowingly introduced language bias and excluded key findings from LMICs and skewed geographic representation. As a result, our synthesis may under-represent perspectives from settings most affected by the challenges under review, and may overlook context-specific barriers, facilitators, or innovations documented outside the English-language literature. Future research with increased capacity and resources for translation could include studies in multiple languages to ensure validity. Several challenges identified in this review such as stigma, limited access, and workforce shortages predate the COVID-19 pandemic, and reflect long-standing structural and societal issues. Whilst these issues are likely to have been exacerbated by the global pandemic, it is difficult to disengage pandemic induced issues from pre-existing ones. We include one study of moderate to low quality in our analysis. However, as we synthesised only the Discussion sections of papers, we do not think low methodological standard has impacted our results. Whilst this approach allowed us to focus on higher-level interpretations across studies, it may have limited the inclusion of rich, firsthand perspectives and potentially emphasized authors' viewpoints over participants' experiences. Although our review identified promising examples of digital health interventions, it is important to note that only 6 of the 30 included studies addressed digital health, limiting the strength of conclusion which can be drawn. Future research is needed to expand the evidence base and to better understand how digital health interventions can be effectively integrated into broader maternity care improvement strategies. An unanswered question remaining for our study is the feasibility and cost-effectiveness of implementing suggested future digital health improvements. We recommend interpretation of qualitative findings with clinical outcomes and health economics.

## 5 Conclusion

This systematic review has the following implications for HCPs and policymakers, for future re-building of maternity services:

1. Improving inclusivity of maternity care, to take into consideration women's emotional challenges, lack of social support systems, and experience of discrimination. Mental health interventions should be implemented routinely as part of maternity care, and digital transformations integrated into existing care pathways to promote continuity and sustainability of systems.
2. Restructuring care delivery systems, to promote community-based care, potentially enabled by digital health solutions, in order to improve accessibility and engagement with care. These should also be utilised to provide clear and consistent public health communication.

However, digital modalities should be evaluated and implemented in a context specific manner, by co-designing with local communities to ensure relevance and acceptability. Sustained education programs to improve health literacy and national investment in digital infrastructure is necessary to mitigate exacerbating inequalities in access.

## Data Availability

The original contributions presented in the study are included in the article/Supplementary Material, further inquiries can be directed to the corresponding author.

## Group members of the RESILIENT study group

Chief Investigator: Prof. Laura A Magee (King's College London); and Co-Investigators: Prof. Debra E Bick (The University of Warwick), Dr. Harriet Boulding (King's College London), Dr. Kathryn Dalrymple (King's College London), Ms. Tisha Dasgupta (King's College London), Prof. Emma L Duncan (King's College London), Dr. Abigail Easter (King's College London), Prof. Julia Fox-Rushby (King's College London), Miss. Gillian Horgan (King's College London), Prof. Asma Khalil (St. George's University Hospitals NHS Foundation Trust & Liverpool Women's NHS Foundation Trust), Ms. Alice McGreevy (King's College London), Dr. Hiten D Mistry (King's College London), Prof. Eugene C Nelson (Dartmouth College), Prof. Lucilla Poston (King's College London), Mr. Paul Seed (King's College London), Dr. Sergio A Silverio (King's College London & University of Liverpool), Dr. Marina Soley-Bori (King's College London), Dr. Florence Tydeman (King's College London), Ms. Aricca D Van Citters (Dartmouth College), Dr. Sara L White (King's College London), Prof. Ingrid Wolfe (King's College London), Prof. Yanzhong Wang (King's College London), & Prof. Peter von Dadelszen (King's College London).
