# Supplementary material for: Seeking digital maternity healthcare during the pandemic health system shock: a systematic review of women's experiences in low- and middle-income countries
Source: Front Reprod Health. 2026 Jan 12;7:1734456. doi: 10.3389/frph.2025.1734456 (PMC12833275; doi:10.3389/frph.2025.1734456)
Supplement: Supplementary file 1 [file Table1.docx]

Supplementary Material

# Supplementary Tables

# Table S1. Search strategy

| Sample | mother OR woman OR women OR midwives OR midwife* OR nurse* OR clinician OR physician OR doctor OR obstetric* OR professional AND |
| --- | --- |
| Phenomenon of Interest | (maternity ADJ care) OR healthcare OR ‘health- care’ OR matern* OR birth* OR childbirth OR pre- nan* OR labour OR labor OR antenatal OR antepa’rtum OR postnatal OR |
|  | postpartum OR post-partum OR puerperium AND coronavirus* OR corona virus* OR COVID-19 OR COVID OR covid OR Covid2019 OR SARS-CoV* OR SARSCov* OR new CoV* OR novel CoV* AND |
| Evaluation and Research Type | E and R: experiences OR experience OR view* OR perceptions OR perception OR voices OR narra- tives OR qualitative OR (mixed ADJ method) OR ‘grounded theory’ OR phenomenology OR ‘action research’. |

# Table S2. Description of included studies

| **Citation** | **Country** | **Study Aim** | **Description of Participants** | **Study dates** | **Data Collection method** | **Data Analysis method** | **Themes identified** | **RESILIENT**  **Theme 1**  **Virtual care** | **RESILIENT**  **Theme 2 self- monitoring** | **RESILIENT**  **Them e 3 vaccination** |  |
| --- | --- | --- | --- | --- | --- | --- | --- | --- | --- | --- | --- |
| Abu Sabbah *et al*. 2022 | Jordan | In this study, we aim to explore and describe expectant mothers’ experiences during the lockdown of COVID-19. | n= 18 Pregnant women with normal pregnancy or with gestational complications that required hospitalization during the pandemic lockdown, and women who have given birth (birth—six weeks) during the lockdown of COVID- 19 were eligible to  participate. | April 30 -  21 June  2020 | phone interviews and a digital demographic survey questionnaire | Thematic analysis | Living with fears and uncertainties amid the COVID-19 Pandemic  Lockdown disrupting the normalcy of pregnancy  Disruption of prenatal care and support  Uncertainty about fulfilling birth plans  Trying to control chaos of life  Taking extra precautions in fear of infection  Seeking reassurance from various available sources  Choosing the 'unusual' for reclaiming control | No | No | No |  |
| Akaba *et al*. 2022 | Nigeria | The objective of the study was to explore the barriers and facilitators of access to MNCH services during the first wave of COVID-19 pandemic in Nigeria. | n=54 across 6 states of Nigeria however only n = 18 were service users.  (it is not made clear in the study what the criteria for service user is) | Between May and July 2020 | In- depth interviews via telephone | Thematic analysis | Delay in seeking care   - socio economic factors - fear of contracting COVID-19 at health facilities Delay in reaching health care facility - Lack of transportation - Movement restriction during the lockdown and harassment by security agents   Delay in receiving care at hospital  - Long waiting times and a daily capped number   - Patient’s non-compliance with the” no- facemask, no entry” rules - Inadequate PPEs - Stigmatization of service users by health workers - Shortage of manpower, lack of incentives and prioritization of essential services   Enablers of decision to seek care   - Community sensitization on healthcare access during the pandemic   Enablers of access to receiving care at the hospital   - •COVID-19 non-pharmacological measures instituted at the health facilities •Adaptive strategies to reduce waiting time at health facilities - Adaptation of service delivery structure and COVID-19 safety protocols •Training and supportive supervision for health workers   Increment in hazard allowance to Health workers | No | No | No |  |
| Akhter *et al*. 2021 | Bangladesh | This study was conducted to explore the lived experience of pregnant women and community health care providers from two southern districts of Bangladesh during the pandemic of COVID-19. | n = 23 of which n = 15 were primi pregnant women or women who had delivered during lockdown) | Between April and June 2020 | In-depth interviews for the pregnant/ postpartum women and Key informant interviews for healthcare providers - both via telephone | Phenomenological approach | Emotional distress   - fear   -worry   - sadness   social and economic distress   - lack of transport - loss of income - difficulty to get a loan   the work environment of the community health work force   - risk of exposure to the virus - hand washing facility   Gendered dimensions and suitability of using PPE - acceptance of PPE suit  - suitability of using the PPE suit during summer | No | No | No |  |
| Ajoke Anokwuru *et al*.2022 | South Africa | The study was part of a larger study that explored the experiences of pregnant women during the COVID- 19 pandemic to develop strategies that can be utilised to assist pregnant women during further pandemics and any type of unforeseen disasters. The purpose of this article is to present the lived experience of utilization of antenatal services among pregnant women in the COVID-19 pandemic. | All women who were 2+ months pregnant and booked for antenatal services around Gauteng and Limpopo province (n=12) | July and August 2020 | In- depth interviews via telephone, zoom or google meet | descriptive e phenomenology | 1. Fear of contracting COVID-19   1a - reduced number of antenatal visits and late bookings  1b - rates of infection in the country 1c - overcrowding at the hospital 1d - use of public transport   1. Limited antenatal services 2a - altered routine services 2b - lack of health education 2. Coping with the new normal   3a - discomfort with wearing of masks 3b - restricted movement   1. Need to digital health education   4a - necessity of having WhatsApp group with midwives  4b need to a specific app for pregnant women's health education | Yes - but only included as future suggestion as oppos ed to use of virtual care in covid pandemic | No | No |  |
| Aşcı *et al*. 2022 | Turkey | The aim of the study was to determine the breastfeeding experiences of COVID- 19-positive women. | n=14 women living in Turkey who had a COVID-19 diagnosis while breastfeeding | 1-25  January 2021 | recorded one-to-one telephone conversation s - semi structured interview | Thematic analysis | Increased emotional load   - feeling sad and inadequate - guilt - anxiety and fear   Breastfeeding during the illness   - refusing treatment and continuing to breastfeed - prioritising mother's milk in feeding the baby/child - difficulty breastfeeding due to physical symptoms - being affected by social media and television   Perceive social support and need   - support by the partner and mother   primary health care providers or health visitors | Yes | No | No |  |
| Aydin *et al*. 2021 | Turkey | This study aimed to investigate the pregnancy experiences of women during the COVID-19 pandemic from the perspectives of pregnant women using a qualitative research method and fill the gap in the relevant field. | n = 14 Pregnant women who were admitted to Karadeniz Technical University Farabi Hospital Pregnancy Outpatient Clinic and were not positive with COVID 19 and over 20 | Dec-20 | Phone interview using descriptive information form and semi- structured in depth interview form | Thematic analysis | Physical health  Psycho-social health   - Anxiety and fear - Depression - Sadness   Adaptation to pregnancy follow-ups   - Postponing follow-ups - Not attending follow-up visits - Preferring health centres that they think are safe   Social life   - Disruption and isolation - Change in social life - The comfort of living in a small province Spousal relationship   Coping methods   - Indoor activities - Focusing on baby   Spousal support | No | No | No |  |
| Aynalem *et al*. 2022 | Ethiopia | to assess the level of COVID-19 vaccine acceptability, determinants, and hesitancy among pregnant mothers attending antenatal care (ANC) at Debre Markos town, public health institutions, Debre Markos, Northwest Ethiopia. | n= 350 pregnant mothers attending ANC at Debre Markos town public health hospitals | sept 1st  - Oct 30th 2021 | Qualitative - unstructured interviewer guide using in depth interview data collection methods | Qualitative = data were analysed under selected themes based on the guide and summarized manually | Reasons for COVID vaccine hesitancy   - rumours - concerns about teratogenicity - effectiveness of vaccine - concern about previous history of drug allergy - adverse effect of vaccine during delivery - unknown effect of vaccine - fear of injection and lack of belief in drug - fear of control via drug - religion related refusal   feeding behaviour protects against COVID | No | No | Yes |  |
| Bankar *et al*. 2022 | India | to under- stand the landscape of health and nutrition services in the times of COVID-19 in March 2020, and a phased re-opening of the same in the selected states thereafter. The data was collected over a period of three months to grasp the status of services and the challenges faced by end users and service providers during the lockdown | n = 12 pregnant women in 2nd or 3rd trimester accessing ANC services (study also included n = 17 frontline health workers) | Not stated | For women - in depth telephone interviews | thematic and narrative analyses | Pregnant women's knowledge and risk perception Anxieties around the pandemic  Access to healthcare services  Service delivery by healthcare functionaries Plans for delivery | No | No | No |  |
| Dewi *et al*. 2021 | Indonesia | The purpose of this study was to explore the anxiety felt by pregnant women holistically about their pregnancy during the COVID-19  pandemic. | n= 20 The informants consisted of 20 pregnant women from three different islands and provinces in Indonesia in February 2021. They were selected to obtain heterogeneous data based on education level, gestational age, urban- rural areas, culture, and religion. | Feb-21 | in depth online interview techniques google and zoom meeting | inductive content analysis | Feeling worried about being infected with COVID- 19 while pregnant  Feeling worried about the impact on health during pregnancy  The impact of the COVID-19 pandemic on the family economy  Circulating information around the community  Unfulfilled spiritual needs in utilizing places of worship | No | No | No |  |
| Draganović *et al*. 2021 | Bosnia | The aim of study was to explore the lived experiences of pregnant women and mothers living in Bosnia and Herzegovina during the COVID-19 outbreak | n= 30 (15 were pregnant and 15 were new mothers) | September and October 2020 | in -depth semi structured interview | interpretive phenomenological analysis | Trapped in the fear of the unknown Adapting and embracing uncertainty | No | No | No |  |
| Freitas-Jesus *et al*. 2022 | Brazil | We aimed to understand the experience of women infected with COVID-19 during pregnancy, regarding their feelings, their relationships, and the influence of social media. | n = 22 women infected with COVID 19 during pregnancy | May-Aug 2020 | (1) semi- directed interviews of open and in- depth questions; (2)  sociodemographic and health data sheets; (3) and field diaries | thematic analysis | (1) The Pandemic Before Infection: the virus existing only on the other side of the world;  (2) Experiences from Diagnosis to Sickness: worrying about the possibilities;  3) The Thing: facing the unnameable virus among close relationships;   1. The Hospitalization and Attachment to the Health Team; and   The Emotional Experience After Recovering from COVID-19 | Yes | No | No |  |
| Dina *et al*. 2021 | Indonesia | To analyze pregnant mother self- empowerment during Covid-19 pandemic in Mustika Jaya District, Bekasi. | n = 4 pregnant mothers | Not stated - paper was published in Jan 2021 | in- depth interviews | Unclear | No specific themes are mentioned in paper That I have identified from reading results:  feeling anxious and worried about pregnancy  - afraid to leave house or go to hospital even when experiencing symptoms | No | No | No |  |
| Lusambili *et al*. 2020 | Kenya | This study aimed to improve understanding of the impact of COVID- 19 on women refugees’ access to and utilisation of antenatal care, delivery and postnatal care in Eastleigh, Kenya. | n = 15 - 10 ANC and 5  PNC mothers (also conducted interviews with healthcare workers so total = 25) | Oct-20 | In depth interviews in person | Thematic analysis | Increase in home deliveries  Reduced facility access to RMNCH services Delayed facility utilisation of RMNCH services  Influences to increase home deliveries, low utilisation and access to RMNCH   - fear and proposed prevention measures - poverty and lack of affordable delivery services   staff negative attitudes and discriminatory policies | No | No | No |  |
| Malik *et al*. 2021 | Pakistan | The present study was designed to evaluate the experiences, perceptions and expectations of pre-birth and post-birth women regarding utilization and delivery of maternal healthcare services in Pakistan. | n = 18 at saturation - Study respondents included pre-birth and post-birth women willing to participate in the study. | can’t find dates when study was conducted - was sent to journal in sept 2021 | semi structured interview | Thematic analysis | 1. Perceptions regarding maternal care services 2. Maternal treatment pathway 3. identified barriers in provision of quality maternal care services 4. choice in healthcare decision making 5. source of knowledge regarding maternal care 6. provision of maternal care counselling 7. impact of COVID on maternal care 8. payments dynamics for maternal care 9. role of digital health in improving maternal care   recommendations for improving maternal care services | Yes | No | No |  |
| Mirzakhani *et al*. 2022 | Iran | The purpose of this study was to explore the experiences of women with high-risk pregnancies who were receiving prenatal care during the COVID-19 pandemic. | n = 31 pregnant women with high-risk pregnancies | Sept 2020 to March 2021. | semi structured interview | qualitative content analysis | Negative psychological responses   - feeling guilty - emotional distress - conflict and doubt   Adjustment of health services in the mutual protection   - access to health services - supportive and protective behaviour   Adoption behaviour   - following health protocols - refusal of health care   coping strategies | Yes | No | No |  |
| Musiimenta *et al*. 2022 | Uganda | This study describes the challenges experienced in accessing maternal and child health services by women with limited or no education during this COVID-19 pandemic and discusses the potential of SNTs to support maternal and child health amidst this crisis. | n= 21 women accessing maternal and child health services (for qualitative part of study) (recruited from previous MatHealth study in 2019 when they were in 1st or 2nd trimester) | Jul-21 | semi structured interview | inductive content analysis | Challenges in accessing health services   - transport to facility - delays at health facility   fear of contracting COVID 19 | Yes | No | No |  |
| Okinarum *et al*. 2022 | Indonesia | to explore breastfeeding experience, such as the strengthening and weakening elements in breastfeeding mothers during the COVID-19 pandemic. | n = 9 postpartum and lactating mothers | between Nov and Dec 2020 | face to face interviews using topic guidelines and open-ended questions | Thematic analysis | Strengthening elements   1. Maternal affection to her baby 2. support system from family and community 3. having adaptive coping strategy Weakening elements 4. impaired comfort 5. insufficient milk supply 6. financial problem 7. parenting problem   indifferent husband | No | No | No |  |
| Paudel *et al*. 2022 | Pakistan | The goal of this study was to understand changes in reproductive, maternal, neonatal, and child health (RMNCH) services during the pandemic in Pakistan | Mix of participants but out of 95, n= 48 were married women of reproductive age with a child under 5 | Nov and Dec 2020 | semi structured in depth interview over telephone | thematic iterative analysis | Changes in care seeking   - Health system disruptions and fear of contracting COVID-19 delays care-seeking - Growing preference for private over public facilities   Changes in RMNCH care provision   - contraception - maternal health - child health - barriers to RMNCH services provision   Suggestions to facilitate RMNCH continuity of care   - increased gov assistance   outreach campaigns | No | No | No |  |
| Paul *et al*. 2022 | Kenya | the aim of this study is to understand how three key audiences–pregnant and lactating women (PLW), male community members, and health workers–in Kenya conceptualize COVID 19 to better understand the determinants of COVID 19 related behaviours | 84 participants of which n=31 were pregnant and lactating women | Aug - Sept 2021 | semi structured interview | grounded theory approach | Myths  risk perception economic implications stigma  self-efficacy | Yes | No | No |  |
| Rauf *et al*. 2021 | Pakistan | This study aims to provide in-depth exploration of the perceptions, fears, and perceived impact of COVID-19 among women with preexisting anxiety symptoms in the late prenatal and postnatal periods in Pakistan. | n = 27 women past 22 weeks gestational age with anxiety | between June and sept 202 | in depth phone interviews | framework analysis | Impact of COVID 19 pandemic   - feeling confined at home - nowhere to deliver the baby - not making ends meet - children losing out on education   Main concerns about COVID 19   - fear of getting infected and infecting newborn - threat to survival - perception of health professionals as damaging   Understanding of COVID 19   - obtaining information about COVID 19 from multiple sources - processing information on COVID 19 | No | No | No |  |
| Reichert *et al*. 2021 | Brazil | To learn the experiences of mothers of infants who were born premature in the Covid- 19 pandemic. | n = 21 mothers of infants who were born premature | Between june and july 2020 | semi structured interview | inductive thematic analysis | Maternal knowledge and perception about COVID 19 and social isolation  Experiences of mothers of premature infants in the COVID 19 pandemic | No | No | No |  |
| Rossetto *et al*. 2021 | Brazil | To understand the repercussions of COVID-19 on the path of pregnancy. | n = 12 pregnant women living in south and southeast regions of Brazil | Oct-20 | Virtual culture circle | Data analysis (themes) was performed concomitantly with the development of the VCC | Flowers in the course of pregnancy Thorns in the course of pregnancy | Yes | No | No |  |
| Sahoo *et al*. 2022 | India | we explored the experiences of antenatal, intra- natal, and postnatal women receiving MCH services in India during the COVID-19 pandemic. | n =48 women either currently pregnant, delivered during the outbreak and those who had a child under 1 year | between April and May 2020 | in depth phone interviews | framework analysis | Antenatal services and nutritional care of pregnant women  Intra-natal and postnatal services Immunisation services (not COVID) Treatment of sick infants | Yes | No | No |  |
| Ulaganeethi *et al*. 2021 | India | To assess the proportion of pregnant women who had not completed the ideal number of antenatal visits, availability of iron–folic acid (IFA) supplements and challenges in availing health services during the period of lockdown. | n=12 pregnant women | 15 July -  7 June  2020 | in depth phone interviews | manual content analysis | Overarching = challenges in availing health services  lock down related health system related psycho-social issues | Yes | No | No |  |
| Pontes *et al*. 2021 | Brazil | the objective of this article is to analyse the affective-semiotic dynamics of women who experienced the transition to motherhood in the Brazilian context of the COVID-19 pandemic from the perspective of Cultural Semiotic Psychology. | n = 8 women who transitioned to motherhood during pandemic | not stated - paper was published in August 2021 | two narrative interviews spaced two months apart | Consensual Qualitative research (CQR)  method | 1 - The perception of personal vulnerability and of the future as uncertain gave rise to the emergence of an affective field characterised by intense fear and anxiety  2 - I-other relations marked by intense ambivalence between the need for social support and the risk of contagion  3 - from this scenario of deep uncertainty and ambivalence, the perspective and experience of childbirth were marked by a strong feeling of insecurity in the relationship with the health contexts, with negative repercussions on the puerperium experience. | No | No | No |  |
| Wang *et al*. 2022 | China | To explore and interpret the experiences of pregnant women in Macao during the COVID-19 pandemic. | n= 18 confirmed as pregnant between Jan and May 2020 | Nov and Dec 2020 | semi structured interview | Thematic content analysis | 1 Changes in daily life   - being confined at home but understanding reasons - financial pressures and timely support from gov   2 Psychological distress   - perceived risk of catching the infection - retaining optimism with various help and support  1. Unique experiences of pregnancy follow-up 2. Trying to pay attention to health information but also feeling overwhelmed 3. change of hygiene behaviours due to fear of infection    - adequate personal protections   obsessive hygiene behaviours | Yes - but only included as future suggestion as oppos ed to use of virtual care in covid pandemic | No | No |  |
| Tilahun Wassie *et al*. 2021 | Ethiopia | to assess pregnant mothers awareness, attitude and practice towards facility childbirth during the covid-19 pandemic and factors related to the non- utilization of facility childbirth services specifically related to community-specific cultural factors | For qualitative study - n = 19 who gave birth in previous 10 months | September 1st- 30th 2020 | Focused group discussion | unclear for qualitative - mention of notes being carefully taken, coded, interpreted | clear themes not illustrated  - results discuss reasons for not attending health facilities, such as cultural beliefs, fear of COVID and the belief giving birth at home is just as good as at a facility | No | No | No |  |
| Widiasih *et al*. 2021 | Indonesia | This study was designed to explore the experiences of pregnant women regarding ANC and monitoring fetal wellbeing during the COVID-19 pandemic. | n = 22 women in their third trimester of pregnancy | November 2020 -  Jan 2021 | semi structured interview | Comparative analysis for interview CAI  approach | 1 Feelings and responses   - positive feelings - negative feelings - response to COVID-19 prevention   2 changes to ANC services during the COVID-19 Pandemic   - wearing PPE - Queues and restrictions based on the number of patients - changes to service hours - the integrated service post provides active services during COVID 19 pandemic   3 Fetal wellbeing monitoring, methods and tools   - motivation for monitoring fetal wellbeing - knowledge of fetal monitoring   methods and tools for fetal monitoring | Yes - but only included as future suggestion as oppos ed to use of virtual care in covid pandemic | Yes | No |  |
| Wolfe-Sherrie *et al*. 2022 | Mexico | (1) Whether postpartum women perceive  COVID-19 as a threat to their and their infants’ health; (2) How the public health mandates affect women’s ability to observe the quarantine;  (3) How the mandates affect postpartum mood. | n = 33 postpartum women | March- December 2020 | telephone interview via survey | content analysis | - Postpartum women’s perceptions of COVID-19 as a threat - COVID-19 public J41health mandates’ effects on the ability to observe the quarantine   COVID-19 public health mandates’ effects on postpartum mood | No | No | No |  |
| **Updated search results (n=1)** | | | | | | | | | | | |
| Amponsah *et al.* 2024 | Ghana | to explore the psychosocial experiences of pregnant women during the COVID-19 pandemic | N=15 nursing mothers, recruited from postnatal clinic who had been pregnant during the pandemic | August 2021 | Semi-structured interviews | Thematic analysis | 2 major themes and 5 subthemes emerged from the study. The unpleasant feelings connected to the potential for contracting COVID-19 and experiencing stress were described by the theme, “Fear and Stress”. Participants’ social experiences (support from significant others), alterations in daily routine and the economic impact because of the pandemic were presented as the “Socioeconomic impact”. | No | No | No |  |

# Table S3. Study quality appraisal

| **Study reference** | **A. Aims and objectives clearly reported** |  | **B. Adequately described the context of research** | **C. Adequately described the sample and sampling methods** | **D. Adequately described the data collection methods** | **E. Adequately described the data analysis methods** | **F. Reliability of the data collection tools** | **G. Validity of the data collection tools** | **H. Reliability of the data analysis** | **I. Validity of the data analysis** | **J. Used the appropriate data collection methods to allow for expression of views** | **K. Used the appropriate methods for ensuring the analysis was grounded in the views** | **L. Actively involved the participants in the design and conduct of the study** |
| --- | --- | --- | --- | --- | --- | --- | --- | --- | --- | --- | --- | --- | --- |
| **Abu Sabbah et al. 2022** | Y |  | Y | Y | Y | Y | Y | Y | Y | Y | Y | Y | N |
| **Akaba et al. 2022** | Y |  | Y | N | Y | Y | Y | Y | Y | Y | Y | Y | Y |
| **Akhter et al. 2021** | Y |  | Y | P | Y | Y | Y | Y | Y | Y | Y | Y | N |
| **Ajoke Anokwuru et al. 2022** | Y |  | Y | Y | P | P | Y | Y | Y | Y | Y | Y | N |
| **Aşcı et al. 2022** | Y |  | Y | Y | Y | Y | Y | Y | Y | Y | Y | Y | N |
| **Aydin et al. 2021** | Y |  | Y | Y | Y | Y | Y | Y | Y | Y | Y | Y | Y |
| **Aynalem et al. 2022** | Y |  | Y | Y | Y | Y | P | P | Y | P | Y | Y | N |
| **Bankar et al. 2022** | P |  | Y | P | Y | Y | Y | Y | Y | Y | Y | Y | N |
| **Dewi et al. 2021** | Y |  | Y | Y | Y | Y | Y | Y | Y | Y | Y | Y | N |
| **Draganović et al. 2021** | Y |  | Y | P | Y | Y | Y | Y | Y | Y | Y | Y | N |
| **Freitas-Jesus et al. 2022** | Y |  | Y | Y | Y | Y | Y | Y | Y | Y | Y | Y | N |
| **Dina et al. 2021** | Y |  | N | N | N | N | N | N | N | N | Y | P | N |
| **Lusambili et al. 2020** | Y |  | Y | P | Y | Y | Y | Y | Y | Y | Y | Y | N |
| **Malik et al. 2021** | Y |  | Y | Y | Y | P | Y | Y | P | P | Y | Y | Y |
| **Mirzakhani et al. 2022** | Y |  | Y | Y | Y | Y | Y | Y | Y | Y | Y | Y | N |
| **Musiimenta et al. 2022** | Y |  | Y | Y | Y | Y | Y | Y | Y | Y | Y | Y | N |
| **Okinarum et al. 2022** | Y |  | Y | Y | Y | Y | Y | Y | Y | Y | Y | Y | N |
| **Paudel et al. 2022** | Y |  | Y | Y | Y | Y | Y | Y | Y | Y | Y | Y | Y |
| **Paul et al. 2022** | Y |  | Y | Y | Y | Y | Y | Y | Y | Y | Y | Y | Y |
| **Rauf et al. 2021** | Y |  | Y | Y | Y | Y | Y | Y | Y | Y | Y | Y | Y |
| **Reichert et al. 2021** | Y |  | Y | Y | Y | Y | Y | Y | Y | Y | Y | Y | N |
| **Rossetto et al. 2021** | Y |  | Y | Y | Y | Y | Y | Y | Y | Y | Y | Y | P |
| **Sahoo et al. 2022** | Y |  | Y | P | Y | Y | Y | Y | Y | Y | Y | Y | N |
| **Ulaganeethi et al. 2021** | Y |  | Y | Y | Y | Y | Y | Y | Y | Y | Y | Y | N |
| **Pontes et al. 2021** | Y |  | Y | P | Y | Y | Y | Y | Y | Y | Y | Y | N |
| **Wang et al. 2022** | Y |  | Y | Y | Y | Y | Y | Y | Y | Y | Y | Y | Y |
| **Tilahun Wassie et al. 2021** | Y |  | Y | Y | P | N | P | P | N | N | P | P | N |
| **Widiasih et al. 2021** | Y |  | Y | Y | Y | Y | Y | Y | Y | Y | Y | Y | Y |
| **Wolfe-Sherrie et al. 2022** | Y |  | Y | Y | Y | Y | Y | Y | Y | Y | Y | Y | N |
| **Amponsah et al. 2024** | Y |  | Y | Y | Y | Y | Y | Y | Y | Y | Y | Y | N |
